# ICESat-2 for Canopy Cover Estimation at Large-Scale on a Cloud-Based Platform

**DOI:** 10.3390/s23073394

**Published:** 2023-03-23

**Authors:** Emre Akturk, Sorin C. Popescu, Lonesome Malambo

**Affiliations:** 1 Department of Ecology and Conservation Biology, Texas A&M University, College Station, TX 77843, USA or eakturk@kastamonu.edu.tr (E.A.); mmoonga@tamu.edu (L.M.); 2 Department of Forest Engineering, Faculty of Forestry, Kastamonu University, Kastamonu 37150, Türkiye

**Keywords:** canopy cover estimation, ICESat-2, ATL08, photon counting lidar, Landsat, Google Earth Engine

## Abstract

Forest canopy cover is an essential biophysical parameter of ecological significance, especially for characterizing woodlands and forests. This research focused on using data from the ICESat-2/ATLAS spaceborne lidar sensor, a photon-counting altimetry system, to map the forest canopy cover over a large country extent. The study proposed a novel approach to compute categorized canopy cover using photon-counting data and available ancillary Landsat images to build the canopy cover model. In addition, this research tested a cloud-mapping platform, the Google Earth Engine (GEE), as an example of a large-scale study. The canopy cover map of the Republic of Türkiye produced from this study has an average accuracy of over 70%. Even though the results were promising, it has been determined that the issues caused by the auxiliary data negatively affect the overall success. Moreover, while GEE offered many benefits, such as user-friendliness and convenience, it had processing limits that posed challenges for large-scale studies. Using weak or strong beams’ segments separately did not show a significant difference in estimating canopy cover. Briefly, this study demonstrates the potential of using photon-counting data and GEE for mapping forest canopy cover at a large scale.

## 1. Introduction

Forest canopy cover is the percentage of the area occupied by the vertical projection of tree crowns in the forest [1,2]. It is a convenient biophysical metric used in various studies related to ecology, biodiversity, forestry, and climate [3,4,5,6]. Canopy cover is an essential ecological property of forests. It is used to determine the presence, condition, diversity, and speed of regeneration of forests besides estimating some critical stand parameters such as Leaf Area Index (LAI) [7,8,9]. Canopy cover also correlates highly with biomass, a vital property of the carbon cycle [10].

Estimating canopy cover for many forestry-related studies has been made with ground-based field sampling measurements. However, using only field measurements, especially in large-scale studies, can cause high costs, biases, uncertainties, and time and labor loss [11,12,13]. In order to minimize these adverse effects in canopy cover estimations, remote sensing technologies have been frequently preferred in many studies. Canopy cover estimates can be efficiently derived by combining field measurements with moderate-resolution satellite images or light detection and ranging (lidar) point cloud data for large regional or global frameworks [14,15,16]. Despite all the advantages of using satellite imagery to estimate canopy cover, relying solely on 2D images can cause some issues because they can be highly affected by topographic and atmospheric conditions, whereas processing lidar data for large-scale areas can be costly as well as storing and analyzing such data can be problematic [17,18]. In addition, there might be regions where ground-based sampling is impossible or very difficult due to field conditions in large-scale studies. At this point, spaceborne lidar instruments offer an excellent opportunity to solve some of the abovementioned issues.

The Ice, Cloud, and land Elevation Satellite (ICESat) was launched by The National Aeronautics and Space Administration (NASA) in 2003, as the first global-scale spaceborne laser altimetry mission [19]. The main objective of this mission was to measure ice sheets and estimate global sea level rise with the help of the Geoscience Laser Altimeter System (GLAS) [20,21]. The first ICESat mission also provided a significant amount of data to analyze terrain, clouds, and vegetation during the mission period [22,23,24]. The ICESat/GLASS system, which provided data on a near-global scale in 33-day periods between 2003 and 2009, was followed by the Advanced Topographic Laser Altimeter System (ATLAS) onboard the ICESat-2 satellite, which was launched in September 2018 [25]. The ATLAS system can make measurements at the photon level, a new laser-based distance estimation technology. Since it is an active system, this technology reduces the operating laser energy and provides a high repetition frequency. Accordingly, the sampling increases considerably [24]. The ICESat-2 laser emits 10,000 light beams per second to the target object and approximately 20 trillion photons in each beam. The system emits six laser beams, three right and left pairs, simultaneously at a distance of 3.3 km from each other. The horizontal distances between beams in the pair are 90 m. Furthermore, the beams are carried in different energies as weak and strong beams with each beam creating a footprint of approximately 14 m in diameter [24,25,26].

The ICESat-2 satellite with its advanced laser altimetry system offers a wealth of information on various biophysical characteristics of the Earth’s surface, including canopy cover. However, the data provided by ICESat-2 are primarily presented in the form of along-track profiles, which may not fully meet the requirements of specific ecological applications that necessitate contiguous maps of canopy cover. In order to overcome this limitation, previous research has employed Landsat satellite imagery as a complementary tool for scaling up or filling in the gaps in the profiling data [27,28,29,30]. This approach has been widely utilized in the scientific community for generating gridded maps of biophysical parameters, such as canopy cover, and has proven to be an effective means of obtaining a more holistic understanding of various ecological processes and patterns. Furthermore, the combination of ICESat-2 and Landsat data allows for the creation of moderate-resolution, multi-temporal datasets that can be used to improve our understanding of the dynamics of ecosystem functioning over time.

Many studies have been carried out in the field of vegetation assessment using ICESat-2 data, which have been providing data since October 2018. It is noteworthy that the majority of these research studies are related to canopy height and biomass estimations [31,32,33,34,35,36,37]. In one of the studies in the literature, besides the aboveground biomass estimation, the canopy cover estimation was performed with the help of photon counting technology [15]. Airborne lidar data were used as the dependent variable for canopy cover estimation, and a regression model was established with simulated ICESat-2 data in that study. As a result of the study, promising R^2^ values ranging from 0.56 to 0.93 were obtained for aboveground biomass and canopy cover estimations. These encouraging results of the canopy cover estimation derived from the study raise some critical questions. The study, as mentioned above, was carried out in a relatively small-scale area of approximately 50 km^2^. Can canopy cover estimation be done efficiently with ATLAS photon data on a larger scale area, for instance, a country or a global scale? Another question is, what success rate can be achieved from the canopy cover estimations when modeled area-wide with the help of satellite images?

Technological developments in recent years are radically changing geospatial computing and thus geospatial analyzes can be conducted easier with such technology as cloud-based platforms. The Google Earth Engine (GEE) service is one of the current examples of such technologies and it provides a multi-petabyte analysis-ready data catalog along with a high-performance parallel computation platform [38]. GEE provides significant convenience in acquiring and analyzing geospatial data, especially in large-scale studies. For this reason, the GEE platform has been preferred in many countries or global-scale studies [39,40,41,42,43].

The ICESat-2 Land/Water Vegetation Elevation (ATL08) product provides terrain and canopy heights in the along-track direction and many other descriptive parameters from the measurements in fixed 100 m. segments [44]. Among the descriptive statistics in the ATL08 product are the number of returned photons in each segment, which is further divided into terrain and canopy photons. Canopy cover can be estimated using the number of photons returned from the ground and canopy, and this information is stored in ATL08 data. As such, the photon count from the canopy would be expected to be higher than the ground since photons cannot reach the ground in an area with dense forest cover. The opposite condition is the case in an area not covered with trees or covered sparsely by vegetation.

In this present article, we would like to address the gap in canopy cover estimation studies by utilizing a cloud-based platform in conjunction with ICESat-2 data to estimate canopy cover at a large-scale, such as at the extent of a country. To the best of our knowledge, this was the first study to employ such an approach at the time of this writing. The proposed methodology has the potential to enhance the efficiency of canopy cover assessments and may have important implications for a wide range of disciplines, including but not limited to forestry, conservation biology, and carbon sequestration. Furthermore, the implementation of a cloud-based platform in this study increases the accessibility and replicability of the data and methods, thereby furthering the potential impact of this study on the scientific community.

The main objectives of this study were to:

1. Develop a methodology to estimate canopy cover with the segments of the ICESat-2 ATL08 product and then test strong or weak energy level beams’ segments separately as inputs in estimation accuracy,
2. Integrate ICESat-2 ATL08 data and satellite imagery to produce a large-scale map using a cloud-based platform, such as GEE,
3. Assess the accuracy of the derived canopy cover map using the visual grid-based accuracy assessment method.

## 2. Materials and Methods

### 2.1. Study Area

**Figure 1:**
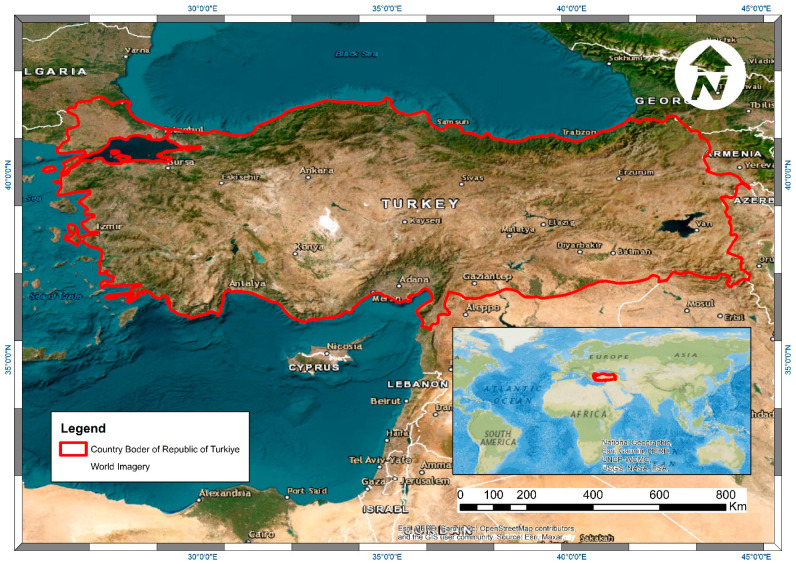
Geographic location and borders of the Republic of Türkiye (study area) (ESRI World Imagery Basemap).

The study area is within the borders of the Republic of Türkiye, with an area of 779,646 km^2^. It is located in the northern hemisphere, between 36–42 north latitudes and 26–45 east longitudes (Figure 1). The country’s location is a bridge between the continents of Europe, Asia, and Africa. This country is in the temperate climate zone since the specified geographical location is closer to the equator than the north pole. Türkiye is one of the richest countries in terms of plant occurrence and diversity of its unique geographical location and climate properties. Official sources state that 29.4% of the country is covered with forest cover in line with the determinations made as of 2020 [45]. Approximately 11,000 naturally grown plant taxa exist, and 35 percent were categorized as endemic taxa [46]. In addition, the study area is located within three phytogeographic regions (Euro-Siberian, Irano-Turanian, and Mediterranean Regions). The dominant vegetation communities of each region are quite different from each other [47,48]. This diversity is an excellent point to demonstrate a study’s effectiveness in estimating the canopy cover.

### 2.2. Data Acquisition and Filtering

This study used the product ICESat-2/ATLAS level 3A ATL08 Version 005 as training and test data for canopy cover estimation. ATL08 datasets in HDF5 format of the study area were downloaded via the NASA Earth Data web-based platform [49]. Due to the canopy cover estimation methodology of this study being based on photon counts, June, July, and August data of 2021, which is known to have forest trees with leaf-on status in the study area, were preferred. After determining the study area borders and dates, a thorough search for appropriate ATL08 tracks was conducted. Eventually, a total of 150 tracks were identified and downloaded, which provided a vast dataset of 2,805,898 segments to work with. These segments were divided into two classes, weak and strong beams, in order to examine whether there is an effect of beam energy in canopy cover estimation. Subsequently, we sorted out the ATL08 variables of interest specified in Table 1 for each segment. The latitude and longitude attributes were used to locate the geographic center of each segment. Pre-labeled canopy, top of the canopy (TOC), and ground photons counts were used to calculate canopy cover by dividing canopy returns by total returns (Equation (1)):

(1) $$Canopy\;Cover=\frac{n\_ca\_photons}{n\_ca\_photons+n\_te\_photons}$$

**Figure 2:**
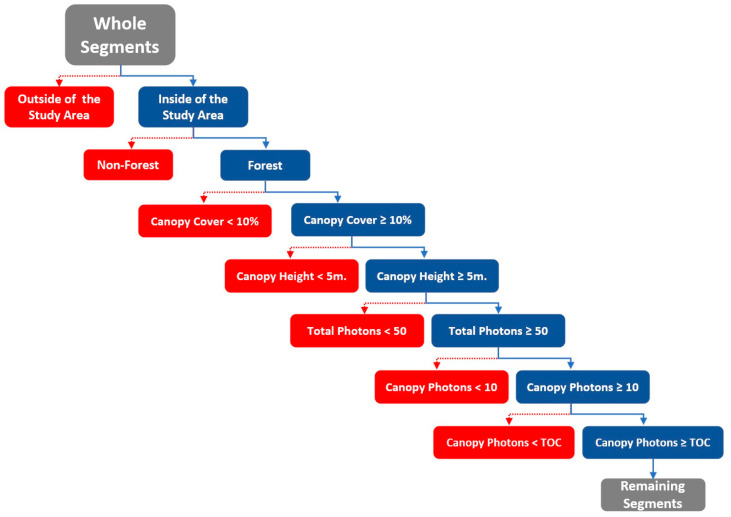
The flow chart depicts the filtering process of ATL08 segments, where red boxes represent filtered segments and blue boxes represent the remaining segments.

‘n_ca_photons’ attribute contains both label 2 (canopy) and 3 (TOC) returns, while ‘n_te_photons’ only covers label 1 (ground) returns in ATL08 version 5. ‘n_toc_photons’ which represents label 3 was used to detect defective segments [44]. The last ‘segment_landcover’ and ‘h_canopy’ data retrieved from the ATL08 data were utilized to determine the land cover of the segment. Since this study was based on estimating the canopy cover of forests, it was decided to use only the segments within the forest cover as training and test data under the forest definition created by Food and Agriculture Organization (FAO) [50], as defined below. With the help of these ATL08 variables, the segments within the study boundaries were filtered in accordance with the following criteria by aiming to use the most suitable segments as the training data set:

- The land cover information of the segments in the ATL08 data was obtained from the 2019 Copernicus Land Cover auxiliary data set with a resolution of 100 m which consists of 23 discrete land cover classes [29,51]. Segments not in the 12 Copernicus Land Cover classes related to forest land cover were excluded from further analysis.
- According to the FAO definition, for an area to be considered a forest, it must have at least 10 percent tree canopy cover, and the tree height must be taller than 5 m. Segments with less than 10 percent canopy cover and tree heights shorter than 5 m were filtered out using the ATL08 variables.
- If a segment has fewer than 50 classified photons, it was accepted as noise and excluded from the analysis. The decision to exclude these segments from the analysis was based on the recommendation provided in the ATL08 product manual [44].
- Similarly, if a segment has fewer than 10 canopy photons, it was accepted as an unreliable segment and filtered out from the dataset [44].
- It was determined that the number of TOC photons in some segments was higher than the number of canopy photons that contains TOC photons beside mid-level canopy photons, and these segments were excluded as errors (Figure 2).

**Figure 3:**
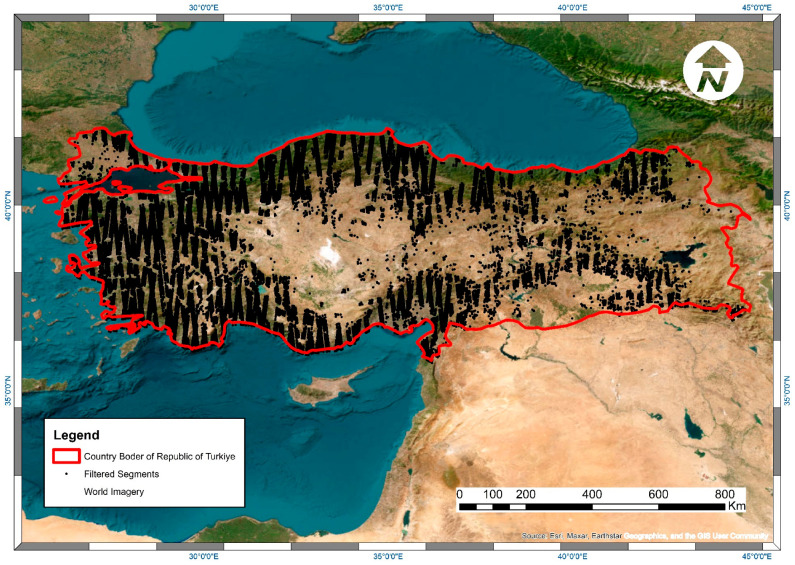
Distribution of filtered segments across the study area (ESRI World Imagery Basemap).

After filtering in line with the above criteria, 446,943 appropriate segments remained for further analysis. The distribution of filtered segments is shown in Figure 3. Calculated canopy cover percentages derived from ATL08 segments were converted into categories based on Turkish Forest Management criteria except to exclude fields with lower than 10 percent canopy cover, which is not accepted as forests under FAO definition [52] (Table 2). Continuing the study with classified canopy cover data also provided avoiding the data processing limits of GEE because of the large scale of the study area.

### 2.3. Canopy Cover Estimation Model (CCEM)

**Figure 4:**
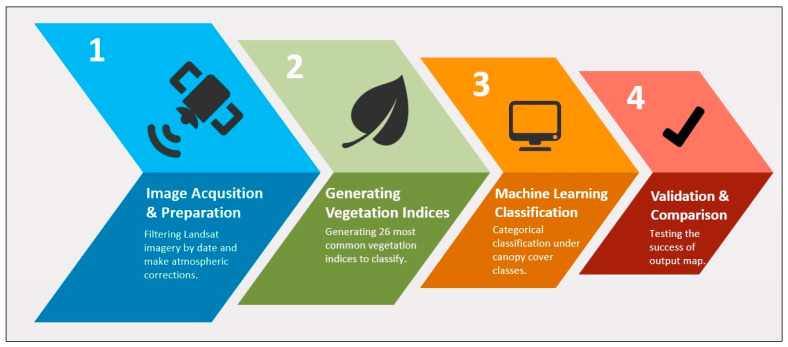
Phases of the canopy cover estimation model.

The model created for canopy cover estimation with ICESat-2 ATL08 data was developed in four phases, and each phase was coded in a JavaScript-based GEE web interface (Figure 4). These phases seem to be the typical steps of image processing techniques, but there were some modifications applied for this study, as noted in the relevant sections.

CCEM is based on the use of spectral features of satellite images besides photon data. For this reason, acquiring appropriate corrected images is of great importance; 368 USGS Landsat 8 Level 2, Collection 2, Tier 1 raw satellite images with 30 m of spatial resolution covering the leaf-on period between 1 June and 31 August 2021, were used within the study area borders. In this study, raw Landsat 8 images were used purposely, and they were processed with the Landsat Simple Composite algorithm in GEE to remove clouds and fill gaps. This algorithm uses the cloud score of each pixel in all satellite images to be assigned with another GEE algorithm called the Simple Landsat Cloud Score algorithm, and then it selects the pixel with the lowest possible cloud score among all images. In this way, a composite image with the lowest possible cloud ratio was obtained for the leaf-on period of the Republic of Türkiye. Spectral bands of the acquired composite image were used to calculate the most common vegetation indices, which are correlated to canopy cover [53,54]. The vegetation indices used are given in Table 3.

First of all, four smaller-scale sample areas (100 × 100 km) were selected within the study area boundaries to assess the efficacy of various vegetation indices in estimating canopy cover. The estimation accuracy of each vegetation index was evaluated, with the Normalized Difference Vegetation Index (NDVI), the Modified Simple Ratio (MSR), and the Green Atmospherically Resistant Index (GARI) demonstrating the highest level of precision at 81.2%, 80.8%, and 80.5%, respectively. To further improve the predictive capability of the selected vegetation indices, they were incorporated in three, five, and seven-band combinations. It was observed that using multiple vegetation indices in combination led to an increase in the estimation accuracy. Notably, an average estimation rate of 84.9% was attained when all vegetation indices were employed. Based on these findings, all indices given in Table 3 were stacked as bands in a single image, and then this image was resampled to 100 m to meet the ATL08 segment size. In addition, pixels under 10 percent canopy cover were masked in this image with the help of the percent vegetation cover for the forest land cover class band within the 2019 Copernicus Land Cover map in order to prevent oncoming issues. The Copernicus Global Land Service (CGLS) is a service that provides a range of bio-geophysical products on the status and evolution of the global land surface. One of its newest products is the Dynamic Land Cover map at 100 m resolution, which offers a global land cover map with 100 m spatial resolution. This product includes continuous field layers for proportional estimates of vegetation/ground cover for different land cover types and provides consistent land cover maps derived from training sites and ancillary datasets. These maps are updated annually with a Sentinel time series [29,51].

The Random Forest classifier was chosen for the categorical classification phase. The Random Forest classifier is a decision tree-based machine learning algorithm commonly used for image classification tasks, which creates multiple decision trees and combines their outputs to produce a final classification result. It is robust to noise and overfitting and provides valuable information such as feature importance [55]. One of the reasons for using this classifier was that the number of GEE-based classifiers that could be used in this study was limited. The other classifier called the Classification and Regression Trees (CART) [56] was tested in this model and the results obtained from the Random Forest classifier were found to be more accurate than CART results. The multi-band vegetation indices image was designated to be a classified image, and the filtered ATL08 segments were used to train this image. Seventy percent of all segments were reserved for image training, and the remaining part was planned for accuracy assessment. Finally, the classifier was run with 128 decision trees to obtain the canopy cover map of the study region.

**Figure 5:**
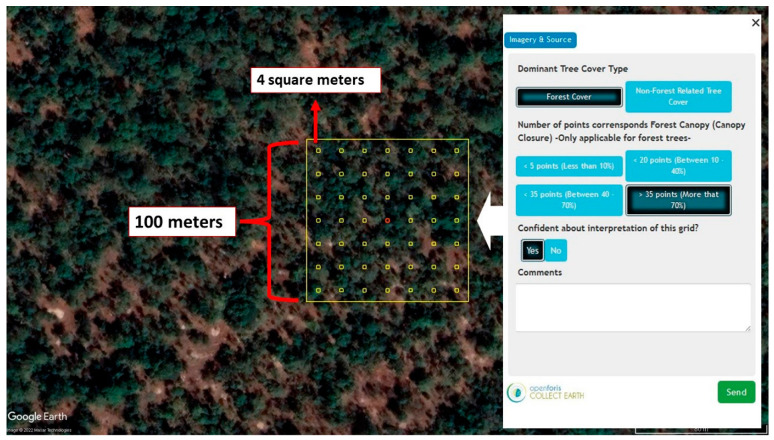
One example of the visual interpretation grids used in this study and the identification card on the right side to collect information about the grid.

Two different methods were employed to assess the accuracy of the obtained canopy cover product. First, an accuracy assessment analysis was performed with 30 percent of filtered ATL08 segments separated from training data. Then, visual interpretations were made at randomly distributed 100-m resolution grids (2000 grids) within the study area with the help of Collect Earth software to validate the CCEM. Collect Earth is a software that enables systematic reference data collection via high-resolution imagery through Google Earth and GEE [57,58]. Each visual interpretation grid consists of 49 small squares of 4 square meters within a 10,000 square meters area which is exactly the same cell size and the geographic location as the pixels of the output canopy cover map (Figure 5). This methodology is based on counting small squares that contain the top of a tree using Google Earth imagery to decide the canopy cover class visually. After the counting process, the necessary information must be entered on the identification card seen in the right part of Figure 5. All this visually gathered canopy cover information was collected in the software, and then the accuracy metrics were calculated with a statistics tool called Saiku Analytics [57]. For a more comprehensive understanding of this accuracy assessment method, interested readers can consult a relevant sample study in the literature [59].

**Table 3:** Calculated vegetation indices in this study to classify canopy cover. Coastal Aerosol, Blue, Green, Red, Near-Infrared (NIR), and Shortwave-Infrared 2 (SWIR2) bands of Landsat 8 OLI were used to calculate these indices.

Vegetation Indices	Source	Formula
Difference Vegetation Index (DVI)	[60]	DVI=NIR−Red
Enhanced Vegetation Index (EVI)	[61]	$EVI=2.5*\;\frac{\left(NIR-Red\right)}{\left(NIR+6*Red-7.5*Blue+1\right)}$
Global Environmental Monitoring Index (GEMI)	[62]	$\;\;GEMI=eta\;\left(1-0.25*eta\right)-\frac{Red-0.125}{1-Red}\;$ $where:\;eta=\;\left(\frac{2\left(NIR^{2}-Red^{2}\right)+1.5*NIR+0.5*Red}{NIR+Red+0.5}\right)\;$
Green Atmospherically Resistant Index (GARI)	[63]	$GARI=\frac{NIR-\left[Green-\gamma \left(Blue-Red\right)\right]}{NIR+\left[Green-\gamma \left(Blue-Red\right)\right]}$
Green Chlorophyll Index (GCI)	[64]	$GCI=\left(\frac{NIR}{Green}\right)-1$
Green Difference Vegetation Index (GDVI)	[65]	GDVI=NIR−Green
Green Leaf Index (GLI)	[66]	$GLI=\frac{\left(Green-Red\right)+\left(Green-Blue\right)}{\left(2*Green\right)+Red+Blue}$
Green Normalized Difference Vegetation Index (GNDVI)	[67]	$GNDVI=\frac{\left(NIR-Green\right)}{\left(NIR+Green\right)}$
Green Optimized Soil Adjusted Vegetation Index (GOSAVI)	[65]	$GOSAVI=\frac{NIR-Green}{NIR+Green+0.16}$
Green Ratio Vegetation Index (GRVI)	[68]	GRVI=NIRGreen
Green Soil Adjusted Vegetation Index (GSAVI)	[65]	$GSAVI=1.5*\frac{\left(NIR-Green\right)}{\left(NIR+Green+0.5\right)}$
Green Vegetation Index (GVI)	[69]	$GVI=\left(-0.2848*CoastalAerosol\right)+\left(-0.2435*Blue\right)+\left(-0.5436*Green\right)+\left(0.7243*Red\right)$ $+\left(0.0840*NIR\right)+\left(-0.1800*SWIR2\right)$
Infrared Percentage Vegetation Index (IPVI)	[70]	$IPVI=\frac{NIR}{NIR+Red}$
Leaf Area Index (LAI)	[71]	$LAI=\left(3.618*EVI-0.118\right)$
Modified Non-Linear Index (MNLI)	[72]	$MNLI=\frac{\left(NIR^{2}-Red\right)*1.5}{NIR^{2}+Red+1.5}$
Modified Soil Adjusted Vegetation Index 2 (MSAVI2)	[73]	$MSAVI2=\frac{2*NIR+1-\sqrt{{\left(2*NIR+1\right)}^{2}-8\left(NIR-Red\right)}}{2}$
Modified Simple Ratio (MSR)	[74]	MSR=NIRRed−1NIRRed+1
Non-Linear Index (NLI)	[75]	$NLI=\frac{NIR^{2}-Red}{NIR^{2}+Red}$
Normalized Difference Vegetation Index (NDVI)	[76]	$NDVI=\frac{\left(NIR-Red\right)}{\left(NIR+Red\right)}$
Optimized Soil Adjusted Vegetation Index (OSAVI)	[77]	$OSAVI=\frac{\left(NIR-Red\right)}{\left(NIR+Red+0.16\right)}$
Renormalized Difference Vegetation Index (RDVI)	[78]	$RDVI=\frac{\left(NIR-Red\right)}{\sqrt{\left(NIR+Red\right)}}$
Soil Adjusted Vegetation Index (SAVI)	[61]	$SAVI=\frac{1.5*\left(NIR-Red\right)}{\left(NIR+Red+0.5\right)}$
Simple Ratio (SR)	[79]	SR=NIRRed
Transformed Difference Vegetation Index (TDVI)	[80]	$TDVI=\;1.5[\frac{\left(NIR-Red\right)}{\sqrt{NIR^{2}+Red+0.5}}]$
Visible Atmospherically Resistant Index (VARI)	[81]	$VARI=\frac{Green-Red}{Green+Red-Blue}$
Wide Dynamic Range Vegetation Index (WDRVI)	[82]	$WDRVI=\frac{\left(0.2*NIR-Red\right)}{\left(0.2*NIR+Red\right)}$

## 3. Results

**Figure 6:**
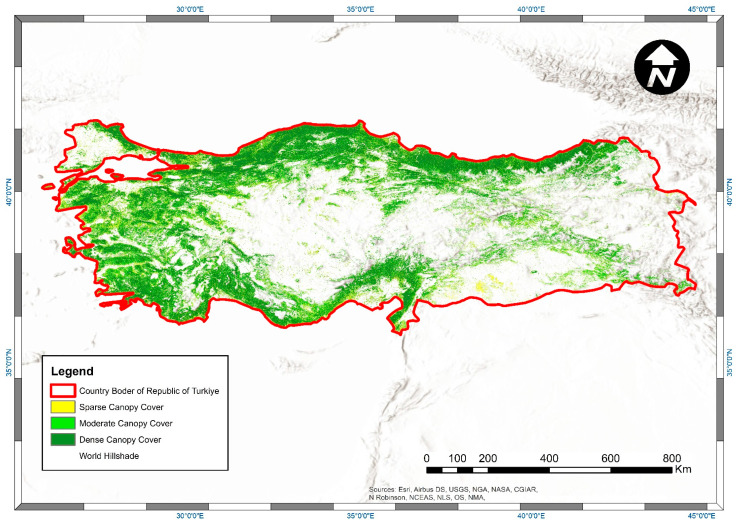
Canopy cover map of Türkiye for the year 2021 derived by ATL08 segments and CCEM (ESRI World Hillshade Basemap).

The canopy cover map of Türkiye for the year 2021 derived from filtered ATL08 segments and CCEM is shown in Figure 6. Results show that 15.2 percent of Türkiye’s forests have sparse canopy cover (between 10 and 40%), 31.7 percent have moderate canopy cover (between 40 and 70%), and the remaining 53.1 percent have dense canopy cover (between 70 and 100%). These results match the inventory work carried out by the General Directorate of Forestry of Türkiye in 2020 [45]. Despite this favorable comparison, discrepancies were found in the country’s total forest area. The total forest land cover on the map obtained from CCEM was found to be approximately 20 percent higher than the forests specified in the country’s inventory studies. This situation affected the accuracy percentages of this study. We determined that the primary source of this issue was the Copernicus Land Cover data, which was used as an auxiliary dataset to mask out other land cover types except for forests. Many agricultural fields or orchards were classified as forests in the Copernicus Land Cover dataset even though it was claimed to have 80.2% overall accuracy [29,51]. This appears to be the main reason why more forested areas were accounted for in this study. Despite the impact on this study, Copernicus Land Cover was used as an auxiliary data set because it has already been used in producing the ATL08 data, and it was the most up-to-date data set on forest areas in GEE for the study period. However, the use of the Dynamic World Land Use/Land Cover (LULC) dataset with a spatial resolution of 10 m, which was recently published and available in the GEE platform, can be tested in this canopy cover estimation model to be updated for the coming years [83].

In the accuracy assessment with the segments reserved for the test, an average accuracy of 79.7% was achieved for CCEM derived canopy cover map of Türkiye, regardless of beam energy. On the other hand, the accuracy assessment performed by canopy cover maps produced separately from segments of weak and strong beams indicated 80.9% and 77.1%, respectively. Although prior research reveals that beams with high energy levels have been more effective in estimating tree height than weak beams, our study yielded contrasting results despite employing a different set of stand parameters. This highlights the need to conduct similar evaluations in diverse study areas to understand better the role of energy levels and other factors in improving the accuracy of canopy cover estimations [84,85]. Because of this, accuracies of canopy cover estimations by segments from different energy levels were also calculated for smaller-scaled sample fields that were previously used to examine the impact of different vegetation indices, and it was confirmed that eliminating any energy level segments from the analysis had no positive effect on estimations. For this reason, all segments were used without considering the energy levels of the segments in the rest of the study.

In addition to the above accuracy assessment, a visual investigation was carried out to assess the accuracy of the canopy cover map generated by CCEM with 2000 interpretation grids. This assessment was conducted with 1928 interpretation grids since the remaining 72 grids did not have high-resolution images to interpret the canopy cover. In addition to counting the squares corresponding to the treetop in the grids, the land cover type was also examined. This way, we aimed to distinguish non-forest fields such as orchards even if there were tree cover on them. The results obtained with the remaining grids are shown in Table 4. The average accuracy in this study was calculated as 71.9 percent with visual interpretations. Average accuracies for canopy cover classes were calculated as 50 percent for SCC, 68 percent for MCC, and 75 percent for DCC. Obtained average accuracy, which can be considered sufficient for a large-scale canopy cover estimation, can be increased to 79.7% if the error in the cover type is not taken into account. This accuracy difference of about eight percent indicates that areas with non-forest trees were not successfully excluded from the analysis. These results are quite remarkable in terms of revealing the issue caused by the auxiliary data set used in the analysis, which was mentioned above.

## 4. Discussion and Conclusions

This study used the ICESat-2 ATL08 product to estimate the canopy cover of a large-scale region at the country level. The accuracy rates above 70 percent indicate that the product obtained as a result of this study can be used as primary or auxiliary data in other studies. These results are auspicious in making canopy cover maps for the coming years and examining vegetation changes over time. As previously stated in this article, only one study in the literature investigated canopy cover estimations using ICESat-2 data during the period when this study was planned [15]. It is important to note that the R^2^ values obtained in that previous study were up to 0.93. However, it would not be appropriate to directly compare the results of the previous study to this current study because the previous study examined an area of 50 km^2^, while this study examined an area that was approximately 15,000 times larger. Furthermore, the present study utilized ICESat-2 ATL08 segments, Landsat 8 satellite imagery, and the Copernicus Land Cover 2019 dataset. Higher success rates would be expected when using ICESat-2 photon data that have been simulated with high-precision lidar data. Using CCEM may produce less precise outputs than the example method, but it can offer significant advantages in reducing time and labor, especially in large-scale studies. As such, researchers who prioritize efficiency and scalability over absolute precision in their canopy cover estimation may find CCEM to be a helpful method.

The CCEM implementation in the GEE platform is not exclusively designed for large-scale studies but can be used for any size of study area. Thus, the CCEM method applies to both small- and large-scale studies. However, an issue for small-scale studies should be considered in CCEM-derived canopy cover products. ATL08 segments are sample data with significant gaps between them. In addition, the number of reflected photons over vegetation is much lower than in other covers due to the nature of photon lidar systems. Especially in small-scale areas, a sufficient number of segments may not be reached to train the data, which may cause bias and uncertainties. In order to solve this problem, multi-year ATL08 datasets can be used as opposed to the one used in this study.

The limitations of the ICESat-2 ATL08 product, which may affect the accuracy of the canopy cover map produced, were acknowledged in the literature [86,87,88]. One of the main limitations is the misclassification of photons in areas with dense vegetation cover, where photons belonging to the terrain may be misclassified as the canopy. However, in this study dense canopies were detected better than other canopy cover classes upon reviewing the results of this study. Additionally, topographic differences in vegetated areas may also influence both the terrain and canopy height metrics of ATL08 between 0.2 m and 2 m. Despite these limitations, the proposed CCEM is still a valuable contribution to the field as it can provide a relatively accurate canopy cover estimation, which is crucial for various ecological applications. Furthermore, it is possible that the limitations of the ATL08 product will be reduced in future versions and then CCEM will produce better estimations with them.

The GEE environment provides many advantages, such as ease of use, no need for upper-level hardware, and the ability to analyze large-scale data relatively quickly. However, especially in large-scale studies, the data processing limits of GEE can be challenging. At the beginning of this study, we planned to establish a model using the canopy cover percentage values obtained from ATL08 with the help of a regression equation instead of a categorical classification method. It was necessary to divide the study area into several small pieces with fishnet to produce a non-categorical canopy cover map of a large-scale area such as the Republic of Türkiye with GEE. In this case, the issue mentioned above of insufficient training segments in small-scale areas may be encountered.

The Copernicus Land Cover map of 2019 was used as an auxiliary dataset in several stages of the study. The most crucial of these stages, the masking out of the non-forest areas from the analysis, could not be carried out with high accuracy, given the confusion with other tree-covered areas that are not forests by definition. This map product has been chosen because it is currently used in the ATL08 product and is one of the most up-to-date land cover maps offered in the GEE platform. There is no doubt that using CCEM with more accurate land cover data will increase the model’s accuracy in future studies.

Landsat satellite images with a spatial resolution of 30 m were used to estimate canopy cover outside of the ICESat-2 tracks. This study can also be carried out with the help of satellite images with higher spatial resolution, such as Sentinel. However, due to the segment size of the ATL08 product corresponding to 100 m resolution, higher resolution satellite images were not preferred in this study. The CCEM can be easily updated and adapted to another segment length of ATL08 when using custom tools such as the PhotonLabeler [89].

The results obtained in this study showed that adequate canopy cover maps could be produced at different scales with the help of the data obtained from the ICESat-2 ATL08 product. Future studies in similar regions or over larger extents could consider incorporating other auxiliary datasets, including data from upcoming missions such as NASA-ISRO Synthetic Aperture Radar (NISAR) [90].

## Figures and Tables

**Table 1:** Selected sub-data for data simplification [44].

ATL08 Data Group	Data Type	Description
latitude	Float	Center latitude of signal photons within each segment
longitude	Float	Center latitude of signal photons within each segment
segment_landcover	Integer	Reference landcover for each segment
n_te_photons	Integer	Number of ground photons within each segment
n_ca_photons	Integer	Number of canopy photons within each segment
n_toc_photons	Integer	Number of TOC photons within each segment
h_canopy	Float	98% canopy height above terrain

**Table 2:** Canopy cover classes based on Turkish forest management practices [52].

Canopy Cover Class	Cover Percentage (%)
Sparse Canopy Cover (SCC)	10–40%
Moderate Canopy Cover (MCC)	40–70%
Dense Canopy Cover (DCC)	70–100%

**Table 4:** Visual accuracy assessment result of CCEM-derived canopy cover map (predicted canopy cover map). The red font in the results indicates the number of interpretation grids that correctly estimated canopy cover class in CCEM derived canopy cover map. The blue font in the results indicates the number of grids that correctly estimated canopy cover class in CCEM derived canopy cover map, but failed in land cover.

		Predicted Canopy Cover Class					
		Forest Tree Canopy Cover			Non-Forest Tree Canopy Cover		
		SCC (10–40%)	MCC (40–70%)	DCC (40–100%)	SCC (10–40%)	MCC (40–70%)	DCC (40–100%)
Actual Canopy Cover Class	SCC (10–40%)	61	42	27	11	7	5
	MCC (40–70%)	6	318	158	1	42	44
	DCC (40–100%)	1	44	1008	1	11	97
	Less Than 10%	1	0	0	40	1	2

## Data Availability

https://code.earthengine.google.com/5b853c4628ec42571826f40b15834dd3 (accessed on 13 February 2023) (Canopy Cover Estimation Model (CCEM) Google Earth Engine (GEE)); This link provides the JavaScript-based code of CCEM prepared for this study within the GEE platform. This link is only accessible for users who register GEE with a Gmail account. This shared code cannot work without training ATL08 segment samples. In order to access training samples; https://code.earthengine.google.com/?asset=users/eakturkphd/Segments/wholePoints (accessed on 13 February 2023) link should be clicked, and the asset must be important to the GEE platform. This asset name should be changed to ‘table’ as stated in the CCEM code before starting to map the canopy cover. These links can also be accessible through the https://figshare.com/s/0f3bd4a9a82b54837921 (accessed on 13 February 2023) link. The codes and data shared here were created by the authors of this article and must not be used without the authors’ permission.
